# Marine invertebrate and seaweed biodiversity of continental coastal Ecuador

**DOI:** 10.3897/BDJ.8.e53818

**Published:** 2020-07-30

**Authors:** Maritza Cárdenas-Calle, Elba Mora, Genoveva Torres, Julián Pérez-Correa, Gregorio Bigatti, Javier Signorelli, Jorge Coronel

**Affiliations:** 1 División Ambiental. Bioelite, Ecuador. Cdla. Bosques el Salado Mz 301 solar 2B frente a Ciudad Colón, Guayaquil, Ecuador División Ambiental. Bioelite, Ecuador. Cdla. Bosques el Salado Mz 301 solar 2B frente a Ciudad Colón Guayaquil Ecuador; 2 Universidad de Guayaquil, Ecuador. Ciudadela Universitaria, Guayaquil, Ecuador Universidad de Guayaquil, Ecuador. Ciudadela Universitaria Guayaquil Ecuador; 3 Universidad de Guayaquil, Ecuador. Ciudadela Universitaria "Salvador Allende", Av. Delta y Av. Kennedy, Guayaquil, Ecuador Universidad de Guayaquil, Ecuador. Ciudadela Universitaria "Salvador Allende", Av. Delta y Av. Kennedy Guayaquil Ecuador; 4 Universidad Espíritu Santo, Ecuador. Km. 2.5 vía La Puntilla,, Samborondón, Ecuador Universidad Espíritu Santo, Ecuador. Km. 2.5 vía La Puntilla, Samborondón Ecuador; 5 LARBIM-IBIOMAR, Centro Nacional Patagónico (CENPAT-CONICET), Puerto Madryn, Argentina LARBIM-IBIOMAR, Centro Nacional Patagónico (CENPAT-CONICET) Puerto Madryn Argentina; 6 Universidad Agraria del Ecuador. Av. 25 de Julio y Pio Jaramillo, Guayaquil, Ecuador Universidad Agraria del Ecuador. Av. 25 de Julio y Pio Jaramillo Guayaquil Ecuador

**Keywords:** Benthos, intertidal rocky shores, subtidal, Ecuadorian coast, marine protected areas.

## Abstract

This study summarises the diversity of living macroinvertebrates and seaweeds from the intertidal and subtidal rocky shores along Ecuadorian continental coast. Benthic macroinvertebrate communities and seaweeds were quantified over quadrants (50 × 50 cm) randomly placed on transects of 50 m length. A checklist of 612 species was generated: 479 species of macroinvertebrates and 133 species of seaweeds. Groups recorded were Mollusca (184 species), Cnidaria (70), Arthropoda (68), Annelida (60), Echinodermata (42), Chordata (18), Bryozoa (13), Porifera (22), Sipuncula (2), Brachiopoda and Platyhelminthes (only identified as morphotypes). The seaweeds were represented by Rhodophyta (78), Chlorophyta (37), Ochrophyta (13), Cyanobacteria (5) and 19 biotic complexes. Furthermore, 22 new taxa and six alien species were recorded from the intertidal zone. This study provides the first large scale report of benthic communities in different marine coastal ecosystems in mainland Ecuador, covering 1,478 km^2^ of protected areas and 382 km^2^ of non-protected areas. The highest benthic diversity was registered in the protected areas and rocky shores from the intertidal zone. The biological data, herein reported, are useful for a long-term monitoring programme to evaluate the status of conservation and to detect rapid changes in the benthic biodiversity from coastal areas.

## Introduction

Biodiversity studies are commonly used to identify changes in the community structure of terrestrial, marine and other aquatic systems and to understand the effects of natural or anthropogenic disturbances on these communities (Cruz-Motta et al. 2010, Vinagre et al. 2016). Managers and scientists are aware of the importance of biological inventories as relevant technical information: to assist sustainable management of biological resources (Drew et al. 2012), to establish baselines for future comparison (Pauly 1995), to expand Marine Protected Areas (MPAs) (Lubchenco et al. 2003), to state biodiversity conservation priorities, to evaluate the environmental quality and health of ecosystems (Edgar et al. 2011), as well as to describe the patterns of biodiversity considering latitudinal gradients (Aued et al. 2018). Changes in biological communities reveal important signals to evaluate the conservation status and the management efficiency of MPAs.

There have been several works carried out on the marine biodiversity in different coastal geographic zones of the south Pacific through expeditions undertaken by European and North American researchers since the 1700s (Olsson 1961). However, most of these surveys have been concentrated in shallow-water and deeper-water down to 200 m depth in Panama, Colombia and Ecuador (Miloslavich et al. 2011). Ecuador is considered an area of high richness of species due to its location in the great Panamic-Pacific zoogeographic province, more precisely from the region extending from Costa Rica southwards to northern Peru (Olsson 1961).

Ecuador has approximately 2,900 km of continental coastline; there is a wide range of geological characteristics, such as bluff, barriers and strand plains, estuaries and lagoons (Boothroyd et al. 1994). Around 1,380 species of invertebrates have been identified in Ecuador, where the Molluscs are the largest group with 110 species. The highest species richness is observed in the southern central coast in the Gulf of Guayaquil (Cruz et al. 2003). There are very few studies of benthic diversity in the rocky shores on the Ecuadorian continental coast. The localities surveyed comprise the north of Ecuador in Galera San Francisco Marine Reserve, Esmeraldas (Reck and Luna 2000), the central coast in Machalílla National Park (Rivera 2012, Ministerio del Ambiente 2015) and the southern central coast in the El Pelado Marine Reserve (Rivera 2011, Cárdenas-Calle et al. 2019, Cárdenas-Calle et al. 2018). The main groups registered in literature in the intertidal and subtidal are Mollusca, Cnidaria, (Rivera et al. 2008); Arthropoda (Mair et al. 2002, Mora et al. 2010, Ministerio del Ambiente 2011, Rivera 2012) and Echinodermata (Mair et al. 2002, Rivera et al. 2008).

Other studies on the coastal zone included a variety of sites along the five coastal provinces of Ecuador recording a total of 140 species of macroinvertebrates including the north (Sua and Punta Galera), central (Puerto Lopez, Los Frailes, Isla de la Plata) and south central shores (Playas, Salinas, Ballenita), including 92 species of molluscs, 31 crustaceans and 17 echinoderms (Mair et al. 2002). Another study was done along 43 sites reporting 527 species in the intertidal zone and 97 species in the subtidal zone (Rivera 2012). All these studies used diverse protocol sampling methods on different spatial and temporal scales. Nevertheless, the spatial distribution of macroinvertebrates associated with rocky shores are similar to other countries near Ecuador in the tropical eastern Pacific, such as Gorgona Island in Colombia, where it was shown that the Mollusca and Crustacea were the most abundant and species rich and where the localities with more irregular topography registered a higher diversity.

The previous studies focused mainly on taxonomic lists, diversity and description of species (Cruz 2004, Cruz 2009, Cruz 2013, Mair et al. 2002, Massay et al. 1993, Mora 1989, Mora 1990, Müller-Gelinek and Salazar 1996, Villamar and Cruz 2007, Villamar 2009, Villamar 2013). Studies on benthic communities from intertidal rocky shores and sandy beaches of Ecuadorian mainland and from zones affected by anthropogenic activity are scarce in literature. Therefore, the objective of this study was to carry out a macrobenthic biodiversity inventory for the intertidal and subtidal zones along the mainland coast of Ecuador.

### Study area descriptions

In order to preserve the marine biodiversity living in the protected areas, the Ecuadorian Government through the Global Environment Funds (GEF) and Inter-American Development Bank (IADB), contributed to update the knowledge of biodiversity in six marine areas (Bioelite 2016). In this work, we report the presence and diversity of marine invertebrates and algae in 10 localities (83 sites) of intertidal and subtidal zones (Tables 1, 2). The study area is extended from Playa Escondida, Esmeralda Province (Lat. 0.818901586 – Long 80.00629363) from the north to Santa Clara Island, El Oro Province (Lat. -3.171890174- Long. 80.4331793) at the south of the Ecuadorian coast, covering 1,478 km^2^ of protected areas and 382 km^2^ of other areas on the mainland coast (Table 1). The protected areas from north to south of the country included were: Galeras San Francisco Marine Reserve (acronym in Spanish: RMGSF) (Esmeralda Province); Wildlife Refuge and Marine Coastal Pacoche (Pacoche) and Machalilla National Park (PNM) at Manabí Province; El Pelado Marine Reserve (acronym: REMAPE) and Wildlife Coastal Marine Reserve Puntilla of Santa Elena (acronym: REMACOPSE) at Santa Elena Province and Santa Clara Island Wildlife Refuge at the El Oro Province. The non-protected areas were: Jama, Canoa at Manabí Province, Ayampe-La Entrada (between Manabí and Santa Elena Provinces) and Cope at Santa Elena Province.

#### North Coast of Ecuador

**Esmeraldas Province**. Galera San Francisco Marine Reserve (RMGSF): this reserve is located in the south of the “Panamic Eco-region” in the southwest of Esmeraldas Province. It was declared a marine reserve in 2008 and has 37 km of coastline. In the marine area, coral reefs and rocky substrates in the subtidal area are observed. On the coast, low cliffs and sandy beaches are predominant. There is an estuarine area where mangroves are present (Ministerio del Ambiente 2014).

#### Central Coast

**Manabí Province.** Machalilla National Park (PNM): this is situated between Jipijapa, Puerto Lopez and Montecristi. It was declared a National Park in 1979. The Humboldt cold current directly affects this area. The National Park is composed of two areas: 1) the terrestrial and 2) the marine area. The latter belongs to the “Guayaquil Eco-region” (Sullivan and Bustamante 1999) and has two types of ecosystems, the marine and the coastal.

Pacoche Wild Life and Marine Reserve (RVSMCP) is located between Manta and Montecristi. Its surface is mainly terrestrial. However, 26468.21 ha are marine coastal environments (Ministerio del Ambiente 2015). The coast is characterised by cliffs, rocky shores, sandy beaches and coral reefs.

Ayampe - La Entrada: This area is not a protected area and is located between Santa Elena and Manabí Provinces. The importance of this area lies in its connectivity with the National Park Machalilla.

Canoa: Located to the north of Caraquez Bay. It is divided into four terrestrial areas of forests.

Jama: It has a surface of approximately 579 km^2^. In the coast cliffs, coral reefs and sandy beaches are predominant. The studied intertidal localities are summarised in Table 1.

**Santa Elena Province.** Puntilla de Santa Elena Marine Faunistic Reproduction Reserve (REMACOPSE): This protected area is located in the Santa Elena Province It was designated as a protected area in 2011. In the marine zone, rocky shores, sandy beaches and mixed substrates have been studied (Ministerio del Ambiente 2011).

El Pelado Marine Reserve (REMAPE): this protected area is also located in Santa Elena Province. It was declared as a Marine Reserve in 2012. Rocky shores, sandy beaches, coral reefs and cliffs are present (Ministerio del Ambiente 2011).

Bajo Cope is an offshore subtidal area located at 15 nautical miles off Montañita (Santa Elena Province). It has 52 km^2^ of total surface and a depth range between 10 and 80 m. A sandy bottom is predominant, but dispersed rocks are observed. This area has been scarcely studied; however, the artisanal and industrial fisheries could affect the entire region. The studied subtidal localities are listed in Table 2.

#### South Coast

**El Oro province.** Santa Clara Island Wild Life Reserve (RVSISC): Santa Clara Island is located in the entrance of Guayaquil Gulf, 43 km west of Puerto Bolívar. It is composed of five islands that are connected at low tide (Hurtado et al. 2010). Santa Clara Island was declared a Natural Protected Areas in 1999.

## Material and methods

**Methodology applied in intertidal studies for sessile and mobile organisms.**

The presence of sessile organisms (macroinvertebrates and seaweeds) in rocky shores was registered following the protocol developed and validated by the group of experts from the "South American Research Group on Coastal Ecosystems (SARCE)" for the sampling of rocky coastlines (SARCE 2012). At each station, three levels of the intertidal levels were studied (high, medium and low), determined according to the dominant biological groups by level. In each level, a transect of 50 m length was applied parallel to the coastline. Over the transect, quadrants (50 × 50 cm) were placed randomly, sampling 30 quadrants per site (10 quadrants for each intertidal level). In each level, the presence of sessile organisms was estimated.

The mobile organisms whose sizes were larger than 1 cm in each quadrant, were identified in the field. The organisms which were not identified, were fixed in 10% formaldehyde and taken as a voucher. Before this, they were relaxed with menthol crystals for two or three hours according to the field guide for specimen collection of the Universidad de Guayaquil (Mair et al. 2000). To register the history of each site, photographs of each quadrant were taken (Rogers et al. 1994).

For sandy beach localities, the methodology used by Aerts et al. (2004) was followed. The fieldwork was undertaken at low tide. Over a transect of 50 m length parallel to the beach line, five quadrants of 50 × 50 cm were placed every 10 m. In each quadrant, the sediment of 10 cm depth was collected and sieved through a 1 mm mesh. Finally, the samples were fixed in seawater with 8% formaldehyde.

**Methodology applied in subtidal studies for sessile organisms and mobile organisms.** Composition of sessile organisms were studied by using a quadrant of 50 × 50 cm and each quadrant was subdivided in 81 intersections. The quadrant was placed every 5 m, along the transect of 50 m length (Edgar et al. 2011). A diver was used to note the taxon or substrate that coincided with each point of intersection. In the cases where the points of intersection did not fall on any organism, only the type of substrate was recorded. The mobile invertebrates (crustaceans, molluscs, echinoderms) were recorded by a second diver on each side of the transect (1 m). The diver registered the presence of species every 5 m.

**Laboratory Analysis.** Mobile and sessile organisms living in the intertidal and subtidal were analysed. During laboratory work, all samples were separated under stereomicroscopes. Taxonomic identification was accomplished through the use of keys and specialised literature for each group such as: for crustaceans (Ball and Haig 1974, Garth 1948, Hickman and Todd 2000, Holthuis 1952); for molluscs (Behrens and Hermosillo 2005, Coan and Valentich-Scott 2012, Giraldo et al. 2014, Keen 1971, Londoño-Cruz et al. 2013, Morris 1966, Olsson 1961); echinoderms (Avilés 1984, Caso 1961, Hendler et al. 1995, Hickman 1998); for corals (Hickman et al. 2005, Hickman 2008); tunicates (De Almeida Rodrigues et al. 1998); seaweeds (Müller-Gelinek and Salazar 1996). The valid name of all the species was corroborated using the WoRMS Editorial Board (2020).

**Figure 1.**
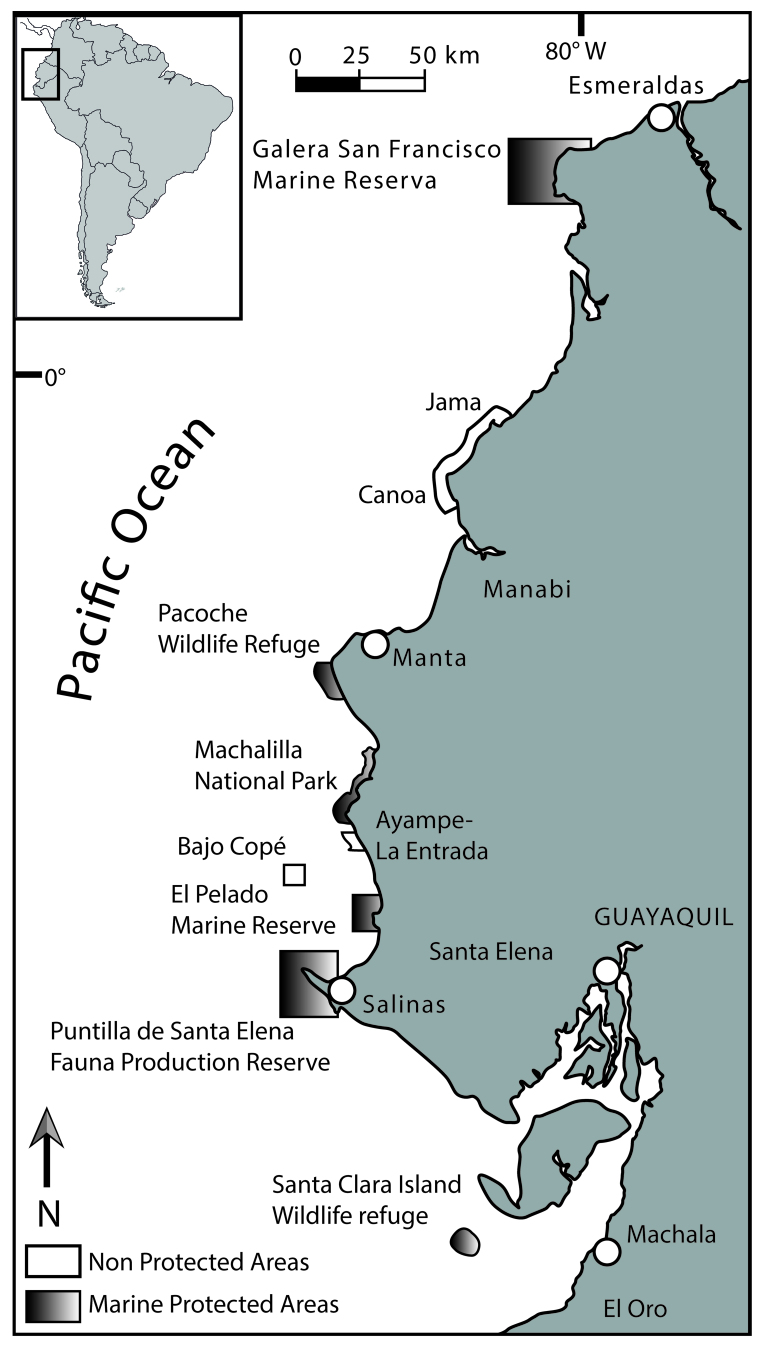
Study area and location of the sampling sites in the Ecuadorian coast.

**General spatial coverage.** The spatial coverage ranged from Lat. 0.818900°; Long -80.006300° at the northernmost site to Lat. -3.189200°; Long -80.452833° at the southernmost site. It encompasses coastal environments of 1860 km (see Fig. 1).

## Results

**Figure 2.**
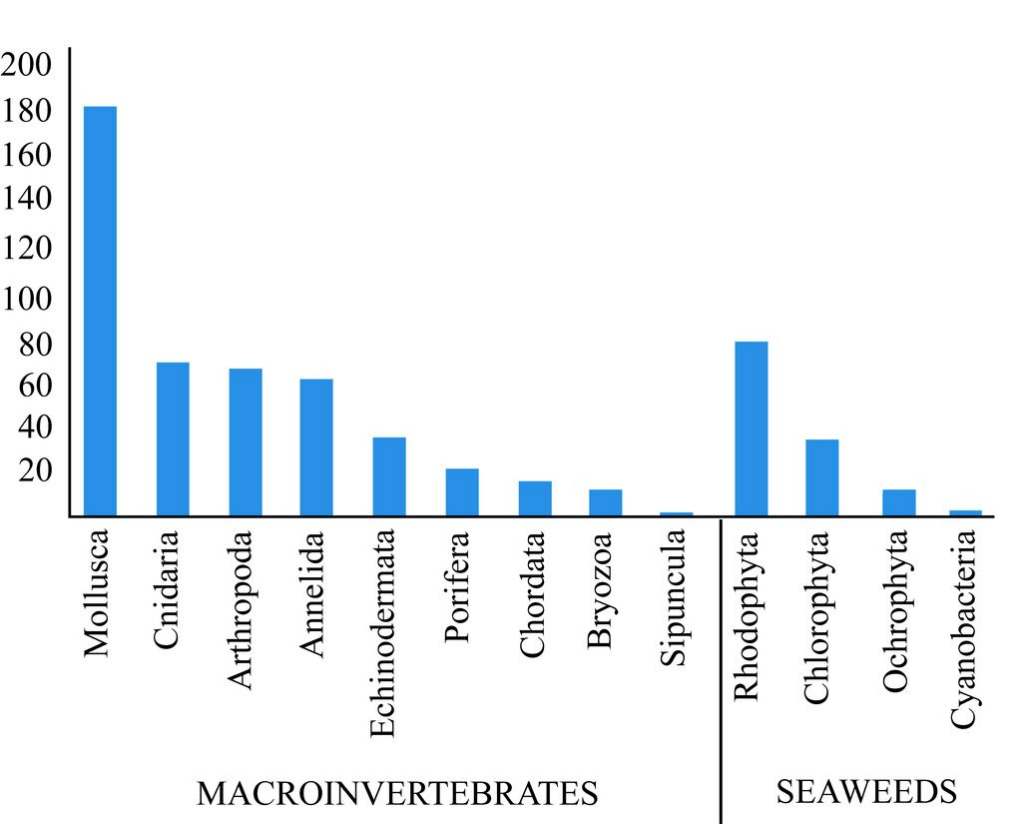
Number of species of macroinvertebrates and seaweeds registered in Ecuadorian coastal during the period 2015-2016.

A total of 83 sites were sampled, 40 in the intertidal zone and 43 in the subtidal zone from protected and non-protected coastal marine coasts (Tables 3, 4, 5, 6). The total taxa identified were 612 corresponding to 479 macroinvertebrates and 133 seaweeds. The determined species belongs to Mollusca (184 species), Arthropoda (68 species), Cnidaria (70 species), Annelida (60 species), Echinodermata (42 species), Porifera (22 species), Urochordata (18 species), Bryozoa (13 species), Sipuncula (two species), Brachiopoda and Platyhelminthes only being identified as morphotype. The seaweeds were represented by Rhodophyta (78 species), Chlorophyta (37 species), Ochrophyta (13 species), Cyanobacteria (five species) and 19 biotic complexes (Fig. 2). The highest biodiversity was registered in the intertidal zone of rocky shores with 423 species. In this zone, the most diverse groups were Mollusca, Annelida, Rhodophyta and Chlorophyta, whereas in the subtidal zone, only 189 species were registered and the most diverse taxa were Rhodophyta, Cnidaria and Echinodermata.

The most common species in the intertidal zone were: *Echinolittorina paytensis*, *E. modesta*, *E. aspera*, *E. porcata*, *Siphonaria palmata*, *Nerita funiculata*, *Fissurella longifissa*, *Anachis rugulosa*, Anachis cf. reedi, *Acanthais brevidentata*, *Vasula melones*, *Olivella semistriata*, *Cerithium gallapaginis*, *Dolabrifera dolafrifera*, *Acanthochitona hirudiniformis*, *Syllis elongata*, *Pareurythoe spirocirrata*, *Pachygrapsus transversus*, *Calcinus obscurus*, *Clibanarius albidigitus* and *Echinometra vanbrunti*. The macroalgae observed with most frequency were *Gelidium pusillum*, *Jania* sp., *Amphiroa* sp., *A. franciscana*, *Polysiphonia bifurcata*, *Boodlea composita*, *Caulerpa racemosa*, *Cladophora* sp. *C. vagabunda*, *Ulva* sp. and *Padina pavonica*.

The mid-tidal and low tidal zones were represented by a variety of macroalgae, polychaetes, echinoderms, molluscs and arthropods, the most frequently observed being: *Nerita funiculata*, *Fissurella longifissa*, *Anachis rugulosa*, Anachis cf reedi, *Acanthais brevidenta*t*a*, *Vasula melones*, *Cerithium gallapaginis*, *Dolabrifera*, *Acantochitona hirudiniformis*, *Syllis elongataPareurythoe spirocirrata*, *Pachygrapsus transversus*, *Clibanarius obscurus*, *C. albidigitus* and *Echinometra vanbrunti*.

In sandy beaches, the most recorded species was *Olivella semistriata*. The highest numbers of species of macroinvertebrates were found in Cabo Pasado and Liguiqui (Manabi Province), Santa Clara Norte (El Oro Province) and Cabo San Francisco in Esmeraldas Provinces. Sessile species were registered mostly in Machalilla (Iturralde and Josse 2000), Pueblo Nuevo and Cabo Pasado (Manabi), Playa Escondida and Cabo San Francisco (Esmeraldas), Santa Clara Sur (El Oro Province), Anconcito and Aqualab (Santa Elena). These results showed a diverse macroinvertebrate community in El Pelado Marine Reserve, Pacoche Wild Life and Marine Reserve and The Cope (Central Coast). The most represented groups were Mollusca, Arthropoda and Seaweeds (Rhodophyta and Chlorophyta) (Tables 3, 4).

In this work, 22 species were registered in the Ecuadorian intertidal zone for the first time: Polychaeta (19), Arthropoda (2) and Mollusca (1). The species were *Paucibranchia oculata*, *Marphysa conferta*, *Oenone* sp., *Maldanella robusta*, *Paleanotus bellis*, *Oxydromus pugettensis*, *Ceratonereis* sp., *Nereis eakini*, *N. vexillosa*, *Platynereis polyscalma*, *Pseudonereis pseudonoodti*, *Perinereis floridana*, *Stenoninereis* sp., *Notophyllum imbricatum*, *Halosydna* sp., *Halosydna johnsoni*, *Lepidasthenia gigas*, *Amblyosyllis* sp., *Asclerocheilus acirratus*, *Joeropsis dubia*, *Paranthura elegans* and *Julia thecaphora*.

The subtidal zone was dominated mainly by sessile organisms, some species with major occurrences being: *Tubastraea coccinea*, *Heterogorgia hickmani*, *Leptogorgia alba*, *Muricea plantaginea*, *M. fruticosa*, *Macrorhynchia philippina* and *Pinctada mazatlanica* while the mobile invertebrates were predominatly *Elysia diomedea*, *Octopus* sp., *Pharia pyramidata*, *Phataria unifascialis*, *Diadema mexicanum*, *Cucumaria flamma*, *Echinometra vanbrunti* and *Eucidaris thouarsii*.

*Taxonomic coverage*. This study recorded 612 species (479 of macroinvertebrates and 133 species of seaweeds). In the intertidal zones, a greater number of species was found (423 species) in relation of subtidal zones (189 species). The most represented groups were Mollusca, Annelida and Rhodophyta (Fig. 2). The Phyla Platyhelminthes and Sipuncula were not identified to species level, but only as morphotype. The highest diversity of mobile macroinvertebrates (323 species) were registered in the intertidal zone, in comparison with the subtidal zone where 157 species of macroinvertebrates (see Tables 3, 5).

## Discussion

The results, herein reported, provide the most recent and extensive baseline study of benthic macroinvertebrates and macroalgae composition in the intertidal and subtidal zones along the Ecuadorian continental coastline from marine protected and non-protected areas. The number of taxa observed in this study was higher in relation to the results reported by Mair et al. (2002) and lower than those registered by Rivera (2012). However, the latter study included in the analysis additional substrates like cracks, stones, beaches and exposed surfaces, while the methodology, herein applied, only included rocky shores and beaches.

The results, herein reported, are the baseline for long term monitoring studies using agile and non-destructive protocols as those used in SARCE and MBON Pole to Pole Projects (SARCE 2012, MBON, P2P 2019). The Molluscs, Cnidarian and Rhodophyta are the main common groups recorded in Ecuadorian coast. More precisely, in the intertidal zone, the gastropods and algae were the more dominant organisms. The invertebrate composition showed a vertical zonation where the low intertidal zone was dominated by *Echinolittorina paytensis*, *E. modesta*, *E. aspera* and *E. porcata*. These results coincide with studies previously performed in Ballenita and Puntilla de Santa Elena and other sites in Ecuadorian coasts, where the family Littorinidae was the most common on the rocky shores (Giraldo et al. 2014, Miloslavich et al. 2013, Miloslavich et al. 2016). The species *Brachidontes playasensis*, *B. adamsianus*, *B. puntarenensis*, *B. semilaevis*, *Chthamalus* sp., *C. panamensis*, *C. southwardorum* and *Jehlius cirratus* are part of a biotic complex in the high intertidal zone. This complex indicates that, besides the physical variation given by desiccation, insolation and thermal stress in the intertidal zone, the incidence of the tide itself contributes with food resources necessary for the survival of grazing gastropods and filtering organism (Littler et al. 1983). Physiological adaptation, such as stomach water storage in gastropods, makes their survival possible in areas with longer drying periods (Herrera-Paz et al. 2013). Nevertheless, sediment and bare rock availability strongly affect the presence of gastropods on the platform (Minchinton and Fels 2013).

The species of commercial interest registered in this study were *Isostichopus fuscus*, *Pollicipes elegans*, *Gelidium pusillum*, *Gigartina* sp., *Centroceras* sp. and *Ulva lactuca* (Chennubhotla et al. 2013, López et al. 2010, Vergara-Chen et al. 2015). In relation to exotic species, six species were recorded (*Amphibalanus amphitrite*, *Pennaria disticha*, *Carijoa riisei*, *Bugula neritina*, *Asparagopsis taxiformis* and *Caulerpa racemosa*). Finally, two bio-indicator species of organically-enriched environments were also registered *Capitella capitata* (Cai et al. 2013) and *Polydora websteri* (Simon and Sato-Okoshi 2015).

This work improves the available information for continental Ecuadorian coasts related to benthic communities living in protected and non-protected areas. It also provides a standardised quantitative report of macroinvertebrates and seaweeds living in the intertidal and subtidal zones. Additionally, this study provides information for ecological and conservation research of marine-coastal environments that has been incipient until the present time, with limited systematised information on biodiversity. However, the scientific study of the marine biodiversity along the Ecuadorian coast remains to be completed as the present survey was developed for areas of special interest to Ecuador’s Ministry of Environment. The available information is especially about commercial species focused on different taxa, such as fishes, crustaceans and molluscs (Coello and Herrera 2010), but is very scarce for the rest of the species. Within this context, the contribution of new research on benthic communities is important to support the country's fishery exports, ensure the sustainability of the food security of Ecuadorians, obtain extraction of active substances for biomedical uses and control the quality of the marine and estuarine ecosystems through bio-indicators of pollution. For this reason, the implementation of a biomonitoring programme is important to compare with other benthic communities and to monitor changes in biodiversity over time by using international standardised methodology.

## Conclusions

The biological data, herein reported, are useful for a long-term monitoring programme to evaluate the status of conservation in protected areas, the influence of anthropogenic factors and the environmental natural changes on the community structure of macroinvertebrates and sessile organisms.

## Figures and Tables

**Table 1.** Intertidal localities and sites on the Ecuadorian continental coast during 2015-2016.

**Localities**	**Site**	**Latitude, Longitude**
Santa Clara Island Wild Life Reserve (RVSISC)	Santa Clara Norte	-3.17097657, -80.4333598
	Santa Clara Sur	-3.17189017, -80.4331793
	Santa Clara Arena	-3.17319399, -80.4351856
Puntilla de Santa Elena Marine Faunistic Reproduction Reserve (REMACOPSE)	Anconcito	-2.33496769, -80.8899295
	Punta Carnero	-2.29432953, -80.9134696
	Punta Carnero Arena	-2.29432953, -80.9134696
	Mar Bravo	-2.26559665, -80.9299928
	Chocolatera	-2.1894211, -81.0088404
	Lobería	-2.20420421, -80.9960879
El Pelado Marine Reserve (REMAPE)	Palmar	-2.02034416, -80.7386037
	Playa Rosada	-2.00748091, -80.7496212
	Aqualab	-1.97160159, -80.7600662
	Playa Bruja	-1.90553499, -80.7301425
Machalilla National Park (PNM)	Salango	-1.59852411, -80.8516345
	La Playita	-1.56522921, -80.8362819
	Puerto López	-1.5458299, -80.8111744
	Pueblo Nuevo	-1.41011648, -80.7540584
	Playa Prieta	-1.48001859, -80.7894161
	Norte de Machalílla	-1.40660621, -80.7544543
Pacoche Wild Life and Marine Reserve (RVSMCP)	Ligüiqui	-1.0212574, -80.8791632
	Cabo San Lorenzo	-1.0562351, -80.9074389
	Santa Rosa Norte	-1.12074148, -80.8890016
	Santa Rosa Sur	-1.12761728, -80.8859992
Galera San Francisco Marine Reserve (RMGSF)	Playa Escondida	0.818901586, -80.0062936
	Punta Galera	0.823950674, -80.0490405
	Estero del Plátano	0.77686593, -80.0893822
	Quingue	0.720880472, -80.0951181
	Cabo San Francisco	0.653120853, -80.0741571
Ayampe - La Entrada	Entrada	-1.72811016, -80.7874128
	Rinconada	-1.71492043, -80.7969534
	Ayampe Norte	-1.68408908, -80.8111077
Canoa	Estero Canoa	-0.45996276, -80.45871
	Canoa Norte	-0.40009256, -80.4929801
	Cabo Pasado	-0.36869868, -80.4764916
	Punta Canoa 1	-0.31378058, -80.4159707
	Punta Canoa 2	-0.34025276, -80.4347689
Jama	Puerto Cabuyal	-0.27614462, -80.3937494
	Puerto Cabuyal Arena	-0.29268238, -80.3976486
	Punta Venado	-0.24981806, -80.3858697
	Punta Ballena	-0.18842368, -80.3099207

**Table 2.** Subtidal localities and sites on the Ecuadorian continental coast during 2015-2016.

**Localities**	**Site**	**Latitude**	**Longitude**
Santa Clara Island Wild Life Reserve (RVSISC)	Sitio 1	-3.18919794, -80.4528352
	Sitio 2	-3.16141917, -80.4422839
	Sitio 3	-3.16237245, -80.4485924
Puntilla de Santa Elena Marine Faunistic	Guarro	-2.29921611, -80.9342183
Reproduction Reserve (REMACOPSE)	Puerto Aguaje	-2.28341051, -80.929902
	Chepan	-2.21404754, -80.9976707
	Gigi María	-2.21316994, -80.9909706
	Casa Lobo	-2.18373044, -81.0036513
	Piedras altas	-2.16662222, -81.0033005
	Bajo Ballena	-2.19635064, -80.957003
El Pelado Marine Reserve (REMAPE)	Pusunga	-1.99387634, -80.7650716
	Bajo 40	-1.93806745, -80.7865788
	Pelado Zona Coral	-1.93578782, -80.7885662
	Planchón	-1.93387932, -80.7921452
	La Pared	-1.93270322, -80.7924241
	Bajo San Ignacio	-1.93154456, -80.7873799
Machalilla National Park (PNM)	Salango	-1.59183003, -80.8640145
	Los Frailes	-1.49324716, -80.8065313
	Horno de Pan	-1.49863047, -80.809012
	Bajo Sucre	-1.47515062, -80.7834475
	Sombrerito	-1.40550411, -80.7705903
Pacoche Wild Life and Marine Reserve (RVSMCP)	Roca Ahogada	-1.01946613, -80.881635
	Roca Ahogada 1	-1.016951, -80.879127
	Cabo San Lorenzo	-1.06021603, -80.911897
Galera San Francisco Marine Reserve (RMGSF)	Piedra de Quingue	0.7276496, -80.1080571
	Tortuga 1	0.76538039, -80.1038528
	Punta Alta	0.65491623, -80.0972692
	Frente al Horno	0.65966567, -80.0983737
Bajo Copé	Seco Manta	-1.81231451, -81.0633161
	Bajo Fer 1	-1.84521958, -81.0527704
	Bajo Fer 2	-1.81640392, -81.0612843
	Bajo Fer 3	-1.81292971, -81.0636668
Ayampe - La Entrada	Rinconada	-1.71222528, -80.8056567
	Rinconada 1	-1.71024404, -80.8066189
	Los Ahorcados 1	-1.6775321, -80.8355716
	Los Ahorcados 2	-1.67766772, -80.8346096
Canoa	Cabo Pasado 1	-0.35703785, -80.4882107
	Cabo Pasado 2	-0.35758954, -80.4850744
	La Saibita	-0.34241556, -80.4449953
Jama	Vaca Brava 1	-0.4070831, -80.3941645
	Vaca Brava 2	-0.23798526, -80.3948835
	Punta Venado	-0.22322058, -80.3879647
	Bajo Londres	-0.17454685, -80.3299424

**Table 3.** Occurrence of mobile macroinvertebrates registered in the intertidal zone in the sampled sites during 2015-2016.

**TAXA**	**RVSISC**	**REMACOPSE**	**REMAPE**	**Ayampe-La Entrada**	**PNM**	**RVSMCP**	**Canoa**	**Jama**	**RMGSF**
										ANNELIDA									
*Pareurythoe spirocirrata* (Essenberg, 1917)	+	+	+	+	+	+			
*Dorvillea moniloceras* (Moore, 1909)		+				+	+		+
*Eunice* sp.							+		
*Lysidice* sp.	+	+					+		+
*Lysidice natalensis* Kinberg, 1865	+								+
*Paucibranchia oculata* (Treadwell, 1921)	+				+				
*Paucibranchia conferta* (Moore, 1911)	+								
*Lumbrineris bassi* Hartman, 1944					+				+
*Scoletoma zonata* (Johnson, 1901)									+
*Arabella iricolor* (Montagu, 1804)	+	+				+	+		
*Diopatra splendidissima* Kinberg, 1865							+		
*Oenone* sp.		+							
*Capitella capitata* (Fabricius, 1780)	+								
*Maldane cristata* Treadwell, 1923	+								
*Maldanella robusta* Moore, 1906							+		+
*Paleanotus bellis* (Johnson, 1897)	+				+	+			+
*Oxydromus pugettensis* (Johnson, 1901)						+			
*Hemipodia* sp.	+								
*Ceratonereis* sp.							+	+	
*Ceratonereis mirabilis* Kinberg, 1865						+			
*Nereis* sp. 1		+	+	+					
*Nereis* sp. 2		+		+					
*Nereis* sp. 3	+				+	+	+	+	
*Nereis* sp. 4					+		+		
*Nereis* sp. 5	+								
*Nereis* sp. 6									+
*Nereis eakini* Hartman, 1936							+		
*Nereis riisei* Grube, 1857						+			+
*Nereis vexillosa* Grube, 1851								+	
*Hediste diversicolor* (Müller, 1776)		+			+				+
*Platynereis polyscalma* Chamberlin, 1919							+		
*Pseudonereis pseudonoodti* (Fauchald, 1977)	+					+			+
*Pseudonereis gallapagensis* Kinberg, 1865					+	+	+	+	+
*Perinereis* sp.					+		+	+	+
*Perinereis floridana* (Ehlers, 1868)	+					+			
*Stenoninereis* sp.	+								
*Aglaophamus verrilli* (McIntosh, 1885)			+						
*Nephtys singularis* Hartman, 1950			+		+		+		
*Notophyllum imbricatum* Moore, 1906		+			+			+	
*Phyllodoce madeirensis* Langerhans, 1880					+	+	+		+
*Halosydna* sp.									+
*Halosydna johnsoni* (Darboux, 1899)									+
*Lepidasthenia gigas* (Johnson, 1897)								+	
*Opisthosyllis arboricola* Hartmann-Schröder, 1959									+
*Syllis elongata* Johnson, 1901	+	+	+	+	+	+	+	+	+
*Syllis* sp.	+								
*Amblyosyllis* sp.	+					+			
*Asclerocheilus acirratus* (Hartman, 1966)									+
ARTHROPODA									
*Neogonodactylus zacae* (Manning, 1972)									+
Amphipoda Latreille, 1816	+	+	+	+	+	+	+		+
Aoridae Stebbing, 1899			+	+			+		
Leucothoidae Dana, 1852			+				+		
Corophiidae Leach, 1814				+					
Gammaridae Leach, 1814		+		+					
Hadzioidea S. Karaman, 1943 Bousfield, 1983	+					+			
Ischyroceridae Stebbing, 1899	+								
Phoxocephalidae G.O. Sars, 1891	+								
Talitridae Rafinesque, 1815		+	+	+			+		
Asellota Latreille, 1802	+								
*Joeropsis dubia* Menzies, 1951	+								
Sphaeromatidae Latreille, 1825	+	+		+	+	+	+	+	+
*Ancinus* sp.	+								
*Dynoides* sp.		+	+				+		
Oniscoidea Latreille, 1802							+		
*Ligia occidentalis* (Dana, 1853)	+	+			+		+		+
Anthuroidea Leach, 1914	+				+		+		+
*Paranthura* sp.	+								
Flabellifera	+				+		+		
Cirolanidae Dana, 1852						+			+
Tanaidacea Dana, 1849	+				+	+		+	+
Alpheidae Rafinesque, 1815	+				+	+	+		+
*Alpheus panamensis* Kingsley, 1878								+	
Upogebiidae Borradaile, 1903							+		
*Upogebia spinigera* (Smith, 1871)							+	+	
*Upogebia tenuipollex* Williams, 1986	+						+	+	+
Anomura MacLeay, 1838								+	
*Coenobita compressus* H. Milne Edwards, 1836					+		+		+
*Calcinus obscurus* Stimpson, 1859		+		+		+	+	+	+
*Clibanarius albidigitus* Nobili, 1901		+			+	+	+	+	+
*Clibanarius lineatus* (H. Milne Edwards, 1848)							+		
*Trizopagurus magnificus* (Bouvier, 1898)		+							
Porcellanidae Haworth, 1825			+		+	+			+
Megalobrachium Stimpson, 1858						+			
*Neopisosoma bicapillatum* Haig, 1960					+	+	+		
*Neopisosoma mexicanum* (Streets, 1871)					+	+	+		+
*Petrolisthes* sp.							+		
*Emerita rathbunae* Schmitt, 1935		+	+				+		
Hippa Fabricius, 1787	+								
Oxyrhyncha	+			+					
Inachiidae									+
*Collodes* sp.	+								
*Microphrys* sp.	+								
*Thoe erosa* Bell, 1835	+						+		
Brachyura postlarvae					+		+		
*Eupleurodon peruvianus* Rathbun, 1924	+								
*Eriphia squamata* Stimpson, 1859						+	+	+	+
*Eriphides hispida* (Stimpson, 1860)							+		
Grapsus Lamarck, 1801	+				+		+		+
*Pachygrapsus transversus* (Gibbes, 1850)	+	+	+	+	+	+	+	+	+
*Geograpsus lividus* (H. Milne Edwards, 1837)		+							
Grapsidae MacLeay, 1838				+		+	+		
*Aratus pisonii* (H. Milne Edwards, 1837)				+					
*Panopeus* sp.	+	+						+	
*Panopeus purpureus* Lockington, 1877							+	+	
*Eurypanopeus planus* (Smith, 1869)								+	
Xanthidae MacLeay, 1838	+				+	+	+	+	+
*Cataleptodius taboganus* (Rathbun, 1912)									+
*Platypodiella rotundata* (Stimpson, 1860)		+							
*Ozius verreauxii* de Saussure, 1853							+		
*Cronius ruber* (Lamarck, 1818)								+	
*Uca* sp.							+		
*Petruca panamensis* Stimpson, 1859							+	+	+
Camptandriidae Stimpson, 1858						+			
MOLLUSCA									
*Donax ecuadorianus* Olsson, 1961			+				+		
*Donax assimilis* Hanley, 1845							+		
*Strigilla dichotoma* (Philippi, 1846)			+						
*Caecum* sp. 1				+		+			+
*Caecum* sp. 2						+			
*Crepidula* sp.						+			
*Crepidula lessonii* (Broderip, 1834)	+								
*Bostrycapulus aculeatus* (Gmelin, 1791)	+								
*Epitonium acapulcanum* Dall, 1917									+
*Echinolittorina* sp. 1		+		+	+				
*Echinolittorina* sp. 2							+		
*Echinolittorina modesta* (Philippi, 1846)	+	+		+	+	+	+	+	
*Echinolittorina aspera* (Philippi, 1846)	+	+	+		+	+	+	+	+
*Echinolittorina porcata* (Philippi, 1846)		+		+	+	+	+	+	+
Echinolittorina cf. albicarinata McLean, 1970				+		+	+		
*Echinolittorina paytensis* (Philippi, 1847)	+	+	+	+	+	+	+	+	+
Rissoina cf. gisna Bartsch, 1915		+							
*Hipponix panamensis* C. B. Adams, 1852				+					
*Hipponix planatus* Carpenter, 1857							+		
*Pilosabia trigona* (Gmelin, 1791)									+
Sinum cf. debile Gould, 1853									+
*Sinum* sp.									+
*Lottia mesoleuca* (Menke, 1851)		+			+		+		+
*Lottia strongiana* (Hertlein, 1958)					+				+
*Lottia subrotundata* (Carpenter, 1865)									+
Lottia cf. dalliana Pilsbry, 1891									+
*Lottia* sp.									+
*Siphonaria* sp.		+			+				
*Siphonaria gigas* G. B. Sowerby I, 1825		+							
*Siphonaria palmata* Carpenter, 1857		+		+	+	+	+		+
*Siphonaria maura* G. B. Sowerby I, 1835		+		+	+				+
*Thylacodes* sp.							+		
*Nerita funiculata* Menke, 1850	+	+		+	+	+	+		+
*Nerita scabricosta* Lamarck, 1822	+	+			+		+		
*Fissurella* sp.		+		+	+				
*Fissurella asperella* G. B. Sowerby I, 1835	+							+	
*Fissurella microtrema* G. B. Sowerby, 1835		+			+	+	+		+
*Fissurella macrotrema* G. B. Sowerby I, 1835	+				+		+		+
*Fissurella longifissa* G. B. Sowerby II, 1862	+	+			+	+	+		+
*Fissurella virescens* G. B. Sowerby, 1835	+	+					+		
*Diodora inaequalis* G. B. Sowerby I, 1835				+					
*Eulithidium phasianella* (Philippi, 1849)		+		+		+	+		+
*Eulithidium umbilicatum* (d'Orbigny, 1840)									+
*Tricolia* sp.							+		
*Pedipes angulatus* C. B. Adams, 1852						+			+
*Cantharus pagodus* (Reeve, 1846)				+					
*Gemophos gemmatus* (Reeve, 1846)	+					+			
*Gemophos ringens* (Reeve, 1846)						+			
*Haplocochlias concepcionensis* (Lowe, 1933)				+					
*Parviturbo* sp.							+		
*Turbo saxosus* Wood, 1828							+		
*Columbella fuscata* G. B. Sowerby I, 1832	+	+					+		
*Columbella major* G. B. Sowerby I, 1832	+								
*Columbella* sp.	+	+							
*Columbella strombiformis* Lamarck, 1822			+				+		
Columbellidae Swainson, 1840		+							
*Anachis* sp.		+					+		
*Anachis fluctuata* (G. B. Sowerby I, 1832)							+		+
Anachis cf. gaskoini Carpenter, 1857				+					
*Anachis lentiginosa* (Hinds, 1844)		+							
*Anachis nigrofusca* Carpenter, 1857						+			
*Parvanachis nigricans* (G. B. Sowerby I, 1844)						+	+		
*Anachis pardalis* (Hinds, 1843)					+	+			
*Parvanachis pygmaea* (G. B. Sowerby I, 1832)						+	+	+	
*Anachis rugulosa* (G. B. Sowerby I, 1844)	+	+		+	+	+	+	+	+
*Anachis rugosa* (G. B. Sowerby I, 1832)									+
Anachis cf. reedi Bartsch, 1928		+	+	+	+	+	+	+	
*Anachis rhodae* Radwin, 1968									+
*Anachis strongi* Bartsch, 1928		+							
*Mazatlania fulgurata* (Philippi, 1846)			+	+	+				+
*Mitrella* sp.				+					
*Mitrella elegans* Dall, 1871						+			
*Mitrella guttata* (G. B. Sowerby I, 1832)	+			+					
*Strigatella tristis* (Broderip, 1836)	+								
*Thais* sp.				+		+	+		+
*Acanthais brevidentata* (Wood, 1828)						+			
*Acanthais callaoensis* (Gray, 1828)	+	+	+	+	+	+	+	+	+
*Acanthais triangularis* (Blainville, 1832)					+				
*Vasula melones* (Duclos, 1832)	+								+
*Trachypollia lugubris* (C. B. Adams, 1852)	+	+	+	+	+	+	+		+
*Stramonita biserialis* (Blainville, 1832)	+								
*Plicopurpura columellaris* (Lamarck, 1816)		+			+	+	+	+	
*Olivella semistriata* (Gray, 1839)							+		
*Cerithium gallapaginis* G. B. Sowerby II, 1855			+	+	+	+	+	+	
*Cerithium nicaraguense* Pilsbry & H. N. Lowe, 1932	+	+		+	+	+	+	+	
*Cerithium* sp.									+
*Bittium* sp.									+
*Bittium peruvianum* (d'Orbigny, 1841)	+				+		+	+	
*Planaxis planicostatus* G. B. Sowerby I, 1825		+							
Planaxis cf. obsoletus Menke, 1851	+				+	+	+		+
*Fossarus guayaquilensis* Bartsch, 1928	+								
*Tegula mariana* (Dall, 1919)						+			
*Pilsbryspira melchersi* (Menke, 1852)	+								
*Onchidella* sp.									+
*Onchidella binneyi* Stearns, 1894		+							
Onchidella cf. steindachneri C. Semper, 1882						+			+
Dorididae Rafinesque, 1815				+	+		+		+
*Julia thecaphora* (Carpenter, 1857)	+								
*Dolabrifera dolabrifera* (Rang, 1828)		+							
*Elysia diomedea* (Bergh, 1894)		+	+		+	+	+		+
*Nudibranchia* sp. 1							+		
*Nudibranchia* sp. 2		+					+		
*Nudibranchia* sp. 3	+				+		+		
*Nudibranchia* sp. 4	+						+		
*Flabellina* sp.									+
Acanthochitona cf. avicula Carpenter, 1857		+							
*Acanthochitona hirudiniformis* (G. B. Sowerby I, 1832)	+								
Acanthochitona cf. hirudiniformis (G. B. Sowerby I, 1832)		+							
Acanthochitona cf. exquisita Pilsbry, 1893				+	+	+	+	+	+
*Acanthochitona* sp.									+
*Chaetopleura* sp.	+								
Tonicia cf. arnheimi Dall, 1903		+							
*Callistochiton elenensis* (G. B. Sowerby I in Broderip & Sowerby, 1832)	+								
*Chiton stokesii* Broderip, 1832	+				+	+	+		
*Ischnochiton dispar* (G. B. Sowerby I, 1832)	+						+		+
*Stenoplax rugulata* (G. B. Sowerby I, 1832)	+					+			
*Ischnochiton* sp.	+								
Polyplacophora Gray, 1821		+	+	+	+		+		+
ECHINODERMATA									
*Echinometra vanbrunti* A. Agassiz, 1863	+	+	+	+	+	+	+		+
*Lanthonia longifissa* (Michelin, 1858)					+				
*Ophiophragmus* sp.									+
*Ophiocomella alexandri* (Lyman, 1860)		+				+			
*Ophiocoma aethiops* Lütken, 1859		+		+	+		+		
*Ophiocoma* sp.		+							
*Ophiothrix* sp.					+				
Ophiothrix (Ophiothrix) spiculata Le Conte, 1851						+			
Hemipholis cf. gracilis Verrill, 1867						+			
*Ophiactis savignyi* (Müller & Troschel, 1842)		+							
Ophiuroidea Gray, 1840	+				+		+		+
Ophiuroidea Gray, 1840									+
Holothuria (Selenkothuria) portovallartensis Caso, 1954							+		
Holothuria (Lessonothuria) pardalis Selenka, 1867							+		
*Isostichopus fuscus* (Ludwig, 1875)	+	+							
Holothuroidea	+						+		
*Heliaster* sp.		+		+	+				
Heliaster cf. cumingi Gray, 1840						+			
*Phataria unifascialis* (Gray, 1840)		+							
*Pharia pyramidata* (Gray, 1840)		+							
*Astropecten cf. armatus* Gray, 1840							+		
SIPUNCULA									
Sipunculidae Rafinesque, 1814		+		+	+	+	+	+	
Sipunculidea sp. 1									+
Sipunculidea sp. 2									+
PLATYHELMINTHES									
Platyhelminthes Minot, 1876	+	+			+		+		+

**Table 4.** Occurrence of sessile invertebrates and seaweeds registered in the intertidal zone in the Ecuadorian coast in nine areas sampled.

Taxa	RVSISC	REMACOPSE	REMAPE	Ayampe-La Entrada	PNM	RVSMCP	Canoa	Jama	RMGSF
PORIFERA									
Porifera sp. 1					+				
Porifera sp. 2		+	+			+			
Porifera sp. 3		+							
Porifera sp. 4	+					+	+		+
Porifera sp. 5									+
Porifera sp. 6									+
Porifera sp. 7	+								
CNIDARIA									
ANTHOZOA									
*Zoanthus cf. pulchellus* Duchassaing & Michelotti, 1860	+	+	+		+		+		+
*Zoanthus* sp. 1		+	+				+		
*Zoanthus* sp. 2							+		
Actiniaria sp 1.	+				+	+		+	
Actiniaria sp 2.						+			
Actiniaria sp 3.						+			
Actiniaria sp 4.						+			
Actiniaria sp 5.						+			
Actiniaria sp 6.							+		
Actiniaria sp 7.							+		
Actiniaria sp 8.									+
Actiniaria sp 9.									+
*Bunodosoma* sp. 1		+							
*Bunodosoma* sp. 2		+			+				
*Bunodosoma* sp. 3				+	+				
*Anthopleura* sp. 1					+		+		+
*Anthopleura* sp. 2									+
Octocorallia Haeckel, 1866	+					+			
*Heterogorgia* sp.						+			
*Tubastraea coccinea* Lesson, 1830						+			
HYDROZOA									
Hydroides Gunnerus, 1768		+							
BRYOZOA									
*Membranipora* sp.						+			
Bryozoa sp.1	+			+	+	+	+	+	+
Bryozoa sp.2	+			+	+				
Bryozoa sp.3						+			
Bryozoa sp.4	+				+	+	+		+
Bryozoa sp.5							+		
Bryozoa sp.6	+								
*Bugula neritina* (Linnaeus, 1758)		+							
BRACHIOPODA									
Brachiopoda Duméril, 1805									+
ANNELIDA									
POLYCHAETA									
*Phragmatopoma californica* (Fewkes, 1889)		+		+	+	+	+	+	+
*Idanthyrsus pennatus* (Peters, 1854)		+		+	+	+			
*Lanice conchilega* (Pallas, 1766)	+				+	+	+	+	+
*Acromegalomma quadrioculatum* (Willey, 1905)	+								
*Boccardia tricuspa* (Hartman, 1939)								+	
*Paraprionospio pinnata* (Ehlers, 1901)				+					
*Polydora websteri* Hartman in Loosanoff & Engle, 1943	+					+	+		+
*Cirratulus cirratus* (O. F. Müller, 1776)	+	+							+
*Tharyx parvus* Berkeley, 1929		+							
*Terebella* sp.	+								
MOLLUSCA									
BIVALVIA									
*Acar rostae* (Berry, 1954)					+	+	+		
*Anadara emarginata* (G. B. Sowerby I, 1833)		+							
*Isognomon janus* Carpenter, 1857	+				+	+	+	+	+
*Sphenia fragilis* H. (Adams & A. Adams, 1854)					+		+	+	+
*Sphenia gulfensis* Coan, 1999							+		+
*Leiosolenus aristatus* (Dillwyn, 1817)	+				+	+	+		+
*Septifer zeteki* Hertlein & A. M. Strong, 1946						+			
*Gregariella coarctata* (Carpenter, 1857)							+		
*Carditamera* sp. Conrad, 1838				+					
*Carditamera radiata* (G. B. Sowerby I, 1833)	+				+	+	+		+
*Carditamera affinis* (G. B. Sowerby I, 1833)		+		+					
*Pseudochama corrugata* (Broderip, 1835)		+							
*Chama* sp.				+			+	+	
*Pholadidea tubifera* (G. B. Sowerby I, 1834)							+	+	+
*Pholadidea melanura* (G. B. Sowerby I, 1834)									+
*Jouannetia pectinata* (Conrad, 1849)									+
*Brachidontes* sp.		+							
*Brachidontes semilaevis* (Menke, 1848)		+			+	+	+	+	
*Brachidontes playasensis* (Pilsbry & Olsson, 1935)	+	+		+	+	+	+	+	+
*Brachidontes adamsianus* (Dunker, 1857)		+		+	+	+	+	+	+
*Brachidontes puntarenensis* (Pilsbry & Lowe, 1932)					+				
*Petricola denticulata* G. B. Sowerby I, 1834				+	+	+	+		+
*Petricola concinna* G. B. Sowerby I, 1834								+	
*Cyrtopleura crucigera* (G. B. Sowerby I, 1834)								+	
*Malleus regula* (Forsskål in Niebuhr, 1775)						+			+
Ostrea cf. conchaphila Carpenter, 1857		+							
*Saccostrea palmula* (Carpenter, 1857)					+				
*Crenella decussata* (Montagu, 1808)		+							
ARTHROPODA									
MAXILLOPODA									
*Pollicipes elegans* (Lesson, 1831)		+							
Balanidae Leach, 1817	+	+				+			+
*Balanus* sp.							+	+	
*Amphibalanus* sp.	+		+	+	+	+		+	
*Amphibalanus amphitrite* (Darwin, 1854)		+		+				+	
*Megabalanus peninsularis* Pilsbry, 1916		+	+	+	+				
Chthamalidae Darwin, 1854		+				+			
*Chthamalus cirratus* Darwin, 1854		+		+	+	+	+	+	+
*Chthamalus* sp. 1						+	+	+	+
*Chthamalus* sp. 2	+						+		
*Chthamalus* sp. 3							+		
*Chthamalus* sp. 4					+	+		+	+
*Chthamalus* sp. 5							+		
*Chthamalus* sp. 6	+				+	+			
*Chthamalus* sp. 7					+				
*Chthamalus* sp. 8	+				+			+	
*Chthamalus* sp. 9	+								
*Chthamalus* sp. 10	+								
*Chthamalus panamensis* Pilsbry, 1916					+	+	+	+	+
*Chthamalus southwardorum* Pitombo & Burton, 2007					+	+		+	+
*Tetraclita squamosa* (Bruguière, 1789)		+		+	+		+		
*Tetraclita* sp.					+	+	+	+	+
Cirripedia Burmeister, 1834					+				
UROCHORDATA:TUNICATA									
Ascidia sp.		+							
Tunicata sp. 1		+			+	+	+		
Tunicata sp. 2		+							
Tunicata sp. 3		+							
Tunicata sp. 4								+	
Tunicata sp. 5								+	
Tunicata sp. 6	+								
*Didemnum* sp.						+			
*Trididemnum* sp.						+			+
BIOTIC COMPLEX									
Complex *Jehlius cirratus* - *Centrocera* sp. - *Ceramium* sp.		+							
Complex *Chthamalus* sp. 4 -*Chthamalus* sp. 8 -*Jehlius cirratus*					+				
Complex *Jehlius cirratus* -Balanidae					+				
Complex *Jehlius cirratus* - *Chthamalus* sp. 4								+	
Complex *Amphinalanus* sp. - *Chthamalus* sp.						+			
Complex *Brachidontes puntarenensis* - *Chthamalus* sp.1					+				
Complex *Brachidontes playasensis* - *Amphibalanus amphitrite*		+							
Complex *Brachidontes playasensis* - *Jhelius cirratus*					+			+	
Complex *Brachidontes playasensis* - *Chthamalus panamensis*								+	
Complex *Brachidontes playasensis* - *Chthamalus* sp. 1								+	
Complex *Ulva clathrata* - *Gelidium pusillum*					+				
Complex *Amphiroa franciscana* - *Ulva lactuca*		+							
Complex *Amphiroa compressa* - *Rhizoclonium crassipelitum*				+					
Complex Rhodophyta - Chlorophyta									+
Complex *Cladophora horii* - *Hypnea cervicornis*								+	
Complex *Boodlea composita* - *Jania capillacea*							+		
Complex *Gelidium* sp. - *Rhizoclonium* sp.							+		
Complex *Jania* sp. - *Asterocystis* sp.	+								
Complex *Jania* sp. - *Ulva* sp. - *Asterocystis* sp.	+								
CYANOBACTERIA									
*Cyanobacteria Stanier* ex Cavalier-Smith, 2002					+			+	
*Oscillatoria* sp. Vaucher ex Gomont, 1892					+		+		
CHLOROPHYTA									
Biofilm Chlorophyta							+		
Chlorophyta sp 1. Pascher, 1914	+								+
Chlorophyta sp 2. Pascher, 1914	+				+				
Chlorophyta sp 3. Pascher, 1914	+						+		
Chlorophyta sp 4. Pascher, 1914	+								
*Boodlea composita* (Harvey) F.Brand, 1904				+	+		+		
*Bryopsis* sp.		+							
*Bryopsis corticulans* Setchell, 1899			+						
*Bryopsis lyngbyei* Hornemann, 1818			+						
*Caulerpa* sp.									+
Caulerpa racemosa (Forsskål) J.Agardh, 1873	+	+					+		
Caulerpa chemnitzia var. laetevirens (Montagne) Fernández-García & Riosmena-Rodriguez, 2017		+							
*Chaetomorpha* sp.				+	+				
*Chaetomorpha antennina* (Bory) Kützing, 1847		+							
*Chaetomorpha minima* F.S.Collins & Hervey, 1917					+				
*Cladophora* sp.		+			+	+	+		+
*Willeella brachyclados* (Montagne) M.J.Wynne, 2016									+
*Pseudocladophora horii* (C.Hoek & Chihara) Boedeker & Leliaert, 2012								+	
*Cladophora panamensis* W.R.Taylor, 1945									+
*Cladophora prolifera* (Roth) Kützing, 1843		+		+					
*Cladophora perpusilla* Skottsberg & Levring, 1941		+							
*Cladophora vagabunda* (Linnaeus) Hoek, 1963		+		+	+				+
*Codium* sp.						+			
*Ulva* sp.					+				
*Ulva flexuosa* Wulfen, 1803		+						+	
*Ulva prolifera* O.F.Müller, 1778		+							
*Ulva clathrata* (Roth) C.Agardh, 1811							+		
*Rhizoclonium* sp. Kützing, 1843					+		+		
*Rhizoclonium crassipellitum* West & G.S.West, 1897				+					
*Spongomorpha conjuncta* W.R.Taylor, 1945							+		
*Struvea* sp. Sonder, 1845		+							+
*Phyllodictyon anastomosans* (Harvey) Kraft & M.J.Wynne, 1996					+				
*Ulva* sp.	+				+	+			+
*Ulva lactuca* Linnaeus, 1753	+	+							
RHODOPHYTA									
Rhodophyta Wettstein, 1901	+					+			+
*Agardhiella subulata* (C.Agardh) Kraft & M.J.Wynne, 1979			+						
*Ahnfeltia* sp.			+						
*Ahnfeltiopsis durvillei* (Bory) P.C.Silva & DeCew, 1992		+							
*Ahnfeltia svensonii* W.R.Taylor, 1945		+							
*Ahnfeltiopsis gigartinoides* (J.Agardh) P.C.Silva & DeCew, 1992		+							
*Amphiroa* sp.			+	+	+	+			+
*Amphiroa beauvoisii* J.V.Lamouroux, 1816					+				
*Amphiroa compressa* M.Lemoine, 1929				+			+		
*Amphiroa franciscana* W.R.Taylor, 1945		+			+		+		
*Scagelia americana* (Harvey) Athanasiadis, 1996		+							
*Chroodactylon* sp.	+								
*Bangia* sp.				+					
*Centroceras* sp. Kützing, 1842 '1841'				+			+		
*Centroceras clavulatum* (C.Agardh) Montagne, 1846			+	+					+
*Ceramium* sp.		+	+	+					
*Ceramium affine* Setchell & N.L.Gardner, 1930					+				
*Gayliella mazoyerae* T.O.Cho, Fredericq & Hommersand, 2008					+				+
*Ceramium dawsonii* A.B.Joly, 1957				+					
*Chrysymenia* sp.			+						
*Corallina* sp.					+		+		+
*Corallina officinalis* Linnaeus, 1758	+			+					
*Erythrotrichia* sp.					+				
*Erythrotrichia carnea* (Dillwyn) J.Agardh, 1883				+			+		
*Erythrotrichia polymorpha* M.A.Howe, 1914		+							
*Gelidium* sp.					+		+	+	+
*Gelidium pusillum* (Stackhouse) Le Jolis, 1863	+	+		+	+	+		+	+
*Endocladia muricata* (Endlicher) J.Agardh, 1847					+				
*Gelidium sclerophyllum* W.R.Taylor, 1945		+		+					+
*Gelidiella* sp.		+	+	+					
*Millerella pannosa* (Feldmann) G.H.Boo & L.Le Gall, 2016		+							+
*Gelidiella machrisiana* E.Y.Dawson, 1957		+							
*Gigartina* sp.		+			+	+	+		
*Chondracanthus acicularis* (Roth) Fredericq, 1993		+							
*Gymnogongrus* sp. Martius, 1833					+				
*Herposiphonia* sp. Nägeli, 1846					+				
*Herposiphonia nuda* Hollenberg, 1968				+					
*Herposiphonia parca* Setchell, 1926									+
*Herposiphonia subdisticha* Okamura, 1899				+			+		
*Herposiphonia tenella* (C.Agardh) Ambronn, 1880		+							
*Hypnea* sp.	+			+	+		+		
*Hypnea spinella* (C.Agardh) Kützing, 1847		+	+		+			+	
*Hypnea pannosa* J.Agardh, 1847		+	+						
*Hypnea valentiae* (Turner) Montagne, 1841		+							
*Hypnea viridis* Papenfuss, 1947			+	+			+		
*Jania* sp.		+	+	+					
*Jania adhaerens* J.V.Lamouroux, 1816			+		+				
*Jania capillacea* Harvey, 1853				+			+		
*Jania pacifica* Areschoug, 1852					+				
*Jania ungulata* Yendo, 1905		+							
*Lithophyllum* sp.	+								+
*Polysiphonia bifurcata* Hollenberg, 1945			+	+	+				
*Polysiphonia howei* Hollenberg, 1945		+							
*Melanothamnus simplex* (Hollenberg) Díaz-Tapia & Maggs, 2017		+	+						+
*Polysiphonia villum* J.Agardh, 1863							+		
*Stylonema* sp.				+					
*Stylonema alsidii* (Zanardini) K.M.Drew, 1956				+					
*Taenioma perpusillum* J.Agardh, 1863				+					
OCHROPHYTA									
Phaeophyceae Kjellman, 1891	+						+		+
*Dictyopteris* sp.			+						
*Dictyopteris delicatula* J.V.Lamouroux, 1809				+					
*Dictyota* sp.							+		
*Hydroclathrus clathratus* (C.Agardh) M.A.Howe, 1920		+					+		
*Padina* sp. Adanson, 1763		+			+				+
*Padina durvillei* Bory Saint-Vincent, 1827			+						
*Padina pavonica* (Linnaeus) Thivy, 1960		+	+				+		
*Padina gymnospora* (Kützing) Sonder, 1871		+							
*Colpomenia* sp.									+
*Colpomenia sinuosa* (Mertens ex Roth) Derbès & Solier, 1851							+		
*Spatoglossum* sp.									+

**Table 5.** Occurrence of taxa from mobile macroinvertebrates registered in the subtidal zone in the Ecuadorian coast in ten areas sampled.

**Taxa**	**RVSISC**	**REMACOPSE**	**REMAPE**	**Ayampe**	**Bajo Cope**	**PNM**	**Pacoche**	**Canoa**	**Jama**	**RMGSF**
MOLLUSCA										
*Conus princeps* Linnaeus, 1758	+									
*Conus rattus* Hwass in Bruguière, 1792								+		
*Conus* sp.									+	
*Monoplex vestitus* (Hinds, 1844)									+	
*Cypraea* sp.					+					
*Latirus* philberti (Récluz, 1844)			+		+		+			
*Polygona concentrica* (Reeve, 1847)								+		
*Pustulatirus mediamericanus* (Hertlein & Strong, 1951)	+						+			
*Pustulatirus sanguineus* (Wood, 1828)						+				
*Latirus* sp. 1				+						
*Latirus* sp. 2				+						
*Opeatostoma pseudodon* (Burrow, 1815)	+				+					
*Triplofusus princeps* (G. B. Sowerby I, 1825)								+		
*Hexaplex brassica* (Lamarck, 1822)		+								
*Hexaplex princeps* Broderip, 1833	+		+			+				
*Hexaplex regius* (Swainson, 1821)										+
*Hexaplex* sp. Perry, 1810		+								
*Vokesimurex elenensis* (Dall, 1909)			+							
*Neorapana muricata* (Broderip, 1832)						+				
*Vasula speciosa* (Valenciennes, 1832)				+						
*Sinum cymba* (Menke, 1828)			+							
*Malea ringens* (Swainson, 1822)	+									
*Lobatus galeatus* (Swainson, 1823)								+		
*Vasum caestus* (Broderip, 1833)										+
*Felimare lapislazuli* (Bertsch & Ferreira, 1974)									+	
*Elysia diomedea* (Bergh, 1894)		+	+	+			+	+		
*Hyotissa solida* Sowerby, 1871			+			+				
*Hyotissa* sp.									+	
*Modiolus eiseni* A. M. Strong & Hertlein, 1937			+							
*Pinctada mazatlanica* Hanley, 1856		+	+	+		+			+	
*Pteria sterna* (Gould, 1851)								+		
*Spondylus crassisquama* Lamarck, 1819				+						
*Periglypta multicostata* (G. B. Sowerby I, 1835)						+				
*Octopus* sp. Cuvier, 1797			+	+	+	+			+	+
ARTHROPODA										
*Stenorhynchus debilis* (Smith, 1871)			+		+	+				
*Neogonodactylus* sp.				+						
*Panulirus gracilis* Streets, 1871	+	+						+	+	+
*Portunus* sp.									+	
ECHINODERMATA										
*Astropecten armatus* Gray, 1840		+								
*Mithrodia bradleyi* Verrill, 1867					+					
*Pharia pyramidata* (Gray, 1840)	+	+	+	+	+	+	+			+
*Phataria unifascialis* (Gray, 1840)	+	+	+	+	+	+	+	+	+	+
*Pentaceraster cumingi* (Gray, 1840)	+	+	+		+	+				
*Nidorellia armata* (Gray, 1840)					+					
*Eucidaris thouarsii* (Agassiz & Desor, 1846)	+	+	+	+	+	+	+	+	+	+
*Astropyga pulvinata* (Lamarck, 1816)			+					+		
*Centrostephanus coronatus* (Verrill, 1867)		+	+		+					
*Diadema mexicanum* A. Agassiz, 1863		+	+		+	+	+			+
*Echinometra vanbrunti* A. Agassiz, 1863		+	+	+	+	+	+			
*Lytechinus semituberculatus* (Valenciennes in Agassiz, 1846)			+							
*Toxopneustes roseus* (Agassiz, 1863)		+		+		+	+			
*Tripneustes depressus* Agassiz, 1863			+	+		+				
*Cucumaria flamma* Solis-Marin & Laguarda-Figueras, 1999	+	+	+	+	+	+	+	+	+	+
*Isostichopus fuscus* (Ludwig, 1875)			+		+	+	+		+	
Holothuria (Thymiosycia) arenicola Semper, 1868								+		
Holothuria (Lessonothuria) pardalis Selenka, 1867		+								
Holothuria (Cystipus) inhabilis Selenka, 1867		+	+			+				
*Ophiocoma aethiops* Lütken, 1859				+				+		
*Ophiocomella alexandri* (Lyman, 1860)							+			
*Ophiocoma* sp.						+		+	+	
*Ophiothrix* sp.							+			
*Ophiothela mirabilis* Verrill, 1867						+			+	

**Table 6.** Occurrence of taxa from sessile invertebrates and seaweeds registered in the subtidal zone in the Ecuadorian coast in ten areas of studies.

**Taxa**	**RVSISC**	**REMACOPSE**	**REMAPE**	**Ayampe**	**Bajo Cope**	**PNM**	**RVSMCP**	**Canoa**	**Jama**	**RMGSF**
PORIFERA										
*Aplysina* sp.		+				+		+	+	
Aplysina cf. chiriquiensis Díaz, van Soest, Rützler & Guzman, 2005										+
*Aplysilla sulfurea* Schulze, 1878			+							
Porifera white incrusting sp. 1		+	+			+				+
Porifera purple sp. 2	+	+	+	+	+	+		+		+
Porifera organge incrusting sp. 3	+	+	+	+	+	+	+	+	+	+
Porifera yellow incrusting sp. 4	+				+					
Porifera gray sp.5					+					
Porifera purple gray sp.6	+									
Porifera black sp.7					+					
Porifera brown incrusting sp.8					+					
Porifera brown purple sp.9										+
Porifera red sp.10					+					
Porifera green sp.11						+				
*Hymeniacidon* sp.										+
*Tethya* sp.		+								
CNIDARIA										
*Palythoa* sp. 1		+								
*Palythoa* sp. 2		+								
*Astrangia* sp.							+			
Caryophyllidae		+	+	+					+	
*Cladopsammia* sp.							+			
*Myriopathes panamensis* (Verrill, 1869)				+						
*Oulangia bradleyi* (Verrill, 1866)			+							+
*Phyllangia* sp.		+								
*Tubastraea coccinea* Lesson, 1830		+	+	+	+	+	+		+	+
*Pocillopora damicornis* (Linnaeus, 1758)			+			+				
*Porites lobata* Dana, 1846							+			
*Pavona gigantea* (Verrill, 1869)				+						
*Carijoa riisei* (Duchassaing & Michelotti, 1860)			+						+	+
*Eugorgia* sp.			+							
*Heterogorgia hickmani* Breedy & Guzman, 2005	+	+	+	+			+		+	+
*Heterogorgia verrucosa* Verrill, 1868				+						+
*Leptogorgia alba* (Duchassaing & Michelotti, 1864)	+	+	+	+		+	+	+	+	
*Leptogorgia cf alba* pink		+	+			+			+	+
*Leptogorgia cuspidata* Verrill, 1865		+						+		
*Leptogorgia laxa* Hickson, 1928		+								
*Leptogorgia pumila* (Verrill, 1868)	+	+								+
Leptogorgia cf. rigida Verrill, 1864										+
Leptogorgia cf. taboguilla Hickson, 1928									+	
*Muricea plantaginea* (Valenciennes, 1846)		+	+	+	+	+			+	+
*Muricea austera* Verrill, 1869	+	+		+	+					+
*Muricea crassa* Verrill, 1869		+							+	+
*Muricea fruticosa* Verrill, 1869	+	+	+	+	+	+	+	+	+	+
*Muricea purpurea* Verrill, 1864	+	+						+		+
*Muricea squarrosa* Verrill, 1869	+	+			+				+	+
*Muricea* sp.								+	+	
*Pacifigorgia adamsii* (Verrill, 1868)	+	+				+	+	+		+
*Pacifigorgia firma* Breedy & Guzman, 2003	+			+	+			+		+
*Pacifigorgia irene* Bayer, 1951				+						+
*Pacifigorgia rubicunda* Breedy & Guzman, 2003	+	+	+							+
*Pacifigorgia stenobrochis* (Valenciennes, 1846)		+		+	+					+
*Psammogorgia* sp.	+	+	+	+						+
*Parazoanthus* sp. 1		+	+	+						+
*Parazoanthus* sp. 2		+	+							
*Parazoanthus* sp. 3		+								
*Parazoanthus* sp. 4						+				
*Palythoa* sp.	+									
*Zoanthus* sp. 1					+					
*Zoanthus* sp. 2	+					+	+			
*Zoanthus* sp. 3	+				+					
*Zoanthus* sp. 4						+				
Hydrozoa Owen, 1843	+				+	+	+			
Dynamena cf. quadridentata (Ellis & Solander, 1786)						+				
*Ectopleura integra* (Fraser, 1938)						+		+		+
*Ectopleura* sp.		+	+							
*Eudendrium* sp.			+							
*Macrorhynchia philippina* Kirchenpauer, 1872	+	+	+	+	+	+	+	+	+	+
*Pennaria disticha* Goldfuss, 1820	+	+		+	+					
*Sertularia turbinata* (Lamouroux, 1816)		+	+	+			+	+		
BRYOZOA										
*Bugulina* sp.		+	+							
*Bugula neritina* (Linnaeus, 1758)		+		+						
*Membranipora membranacea* (Linnaeus, 1767)			+							
Bryozoa purple encrusting				+						
*Plesiocleidochasma porcellanum* (Busk, 1860)										+
ANNELIDA										
*Idanthyrsus pennatus* (Peters, 1854)				+						
*Phragmatopoma californica* Fewkes, 1889	+				+					
Sabellariidae Johnston, 1865	+		+		+		+			+
*Serpula* sp.						+		+		
*Spirobranchus giganteus* (Pallas, 1766)		+	+	+		+	+	+		+
Polychaeta Grube, 1850										+
Polychaeta tube-dwelling		+								
MOLLUSCA										
*Cryptomya californica* (Conrad, 1837)			+							
*Hyotissa fisheri* (Dall, 1914)							+			
*Pecten* sp.	+									
*Pinctada mazatlanica* (Hanley, 1856)						+				
*Hexaplex princeps* (Broderip, 1833)			+							
ARTHROPODA: MAXILLOPODA										
*Balanus* sp. 1		+	+			+				
*Balanus* sp. 2			+							
*Balanus trigonus* Darwin, 1854			+							
*Megabalanus peninsularis* (Pilsbry, 1916)							+			
*Tetraclita* sp.		+								
CYANOBACTERIA										
Cyanophyceae sp. 1 Schaffner, 1909	+		+				+		+	+
Cyanophyceae sp. 2 Schaffner, 1909						+		+	+	
*Lyngbya majuscula* Harvey ex Gomont, 1892						+				
*Oscillatoria* sp.						+				
OCHROPHYTA										
Phaeophyceae Kjellman, 1891		+								
*Dictyota* sp.	+	+	+		+	+		+		
*Padina* sp.	+	+	+	+		+				
RHODOPHYTA										
Rhodophyta sp. 1		+	+	+						
Rhodophyta sp. 2	+	+	+			+				+
Rhodophyta sp. 3	+		+							
Rhodymeniales Schmitz in Engler, 1892			+							
*Acanthophora* sp.		+								
*Amphiroa* sp.	+	+				+				
*Asparagopsis* sp.	+									
*Asparagopsis taxiformis* (Delile) Trevisan de Saint-Léon, 1845			+							
*Chondria* sp.			+			+				
Corallinaceae Lamouroux, 1812			+			+		+	+	+
Corallina cf. officinalis Linnaeus, 1758		+								
*Galaxaura* sp.			+							
*Gelidium* sp.			+			+				
*Peyssonnelia rubra* (Greville) J.Agardh, 1851					+					
*Ceratodictyon* sp.	+									
*Gracilaria* sp.			+							
*Hildenbrandia rubra* (Sommerfelt) Meneghini, 1841		+			+					
*Hildenbrandia* sp.		+	+							+
*Hypnea* sp.		+								
*Liagora* sp.		+								
*Lithophyllum* sp.	+	+	+	+	+		+			+
*Martensia* sp.	+		+							
*Laurencia* sp.	+	+	+							
CHLOROPHYTA										
Chlorophyta Pascher, 1914					+	+				
Chlorophyta (filamentous) Pascher, 1914							+	+		+
*Bryopsis* sp.		+								
*Caulerpa chemnitzia* (Esper) J.V.Lamouroux, 1809										
*Codium* sp.	+	+								
*Ulva* sp.		+	+			+				
*Valonia* sp.										
UROCHORDATA: TUNICATA										
*Aplidium* sp.					+					
*Trididemnum* sp. 1					+					
*Trididemnum* sp. 2								+		
*Ascidia* sp.						+				
*Clavelina* sp.	+		+							
*Eudistoma* sp.					+					
Didemnum cf. vexillum Kott, 2002						+				
*Didemnum* sp. 1 (white)		+	+							
*Didemnum* sp. 2 (purple)			+							
